# Pomgulated methionil (LY2140023) in schizophrenia patients: a systematic review and meta-analysis

**DOI:** 10.1186/s12888-025-07199-z

**Published:** 2025-08-08

**Authors:** Hala Aboushawareb, Omar F. Abbas, Hend Ghabour, Omar Hassan, Ali Nagy Shelbaya, Rahma Abdelsalam, Mostafa Meshref

**Affiliations:** 1 Faculty of medicine, Mansoura university, Mansoura, Egypt; 2 Faculty of medicine, Al-Azhar university, Cairo, Egypt; 3 Helwan psychiatric Hospital, Egyptian ministry of health, Cairo, Egypt; 4 Faculty of Medicine, Cairo University, Cairo, Egypt; 5 Faculty of Medicine, New Mansoura University, Manosoura, Egypt; 6 Department of Neurology, Faculty of Medicine, Al-Azhar University, Cairo, Egypt

## Abstract

**Background:**

Pomaglumetad methionil (LY2140023 monohydrate) is a potent and selective agonist for metabotropic glutamate receptors (mGluR2/3). Unlike traditional antipsychotics, it does not directly interact with dopamine or serotonin (5-HT2A) receptors, potentially offering a novel mechanism of action with a different side-effect profile. We aim to provide an overview of this novel drug and evaluate its efficacy in comparison to both placebo and atypical antipsychotics by performing a systematic review and meta-analysis.

**Methods:**

A comprehensive literature search was conducted to identify relevant studies. The included studies investigated the effect of Promogulated methionil. The quality of studies was assessed using the Cochrane Risk of Bias 2 (ROB-2) Statistical analysis was conducted using Review Manager (revman) with outcomes expressed as Mean differences (MD) with 95% confidence intervals (CI).

**Results:**

The systematic review included 4 randomized clinical trials (RCTs). The analysis revealed that pomaglumetad methionil (LY2140023) didn’t have a statistically significant effect on PANNS compared to placebo (p-value = 0.31) and it was less effective in decreasing PANSS score in comparison to atypical antipsychotics (p-value < 0.00001). However, the drug showed a significant effect on weight gain (p-value < 0.00001) and prolactin (*p* < 0.0001) in comparison to atypical antipsychotics.

**Conclusions:**

In conclusion, this systematic review and meta-analysis provide evidence that pomaglumetad methionil (LY2140023) does not demonstrate consistent efficacy in the treatment of schizophrenia. Although the compound is associated with a more favorable profile regarding weight gain and prolactin elevation, these advantages do not compensate for its lack of therapeutic efficacy.

## Introduction

Schizophrenia is a chronic and debilitating mental disorder that affects approximately 24 million people globally [1]. Schizophrenia is ranked among the top 10 causes of disability globally, as it impairs individuals’ ability to perform basic social and functional tasks [2]. Many patients experience profound difficulties in forming social relationships, while retaining comparatively less impairment in self-care and physiological functions. The manifestation of disability in schizophrenia is largely influenced by symptomatology [3], which varies across patients.

Clinically, schizophrenia is characterized by positive symptoms, negative symptoms, and cognitive deficits [4]. Positive symptoms include perceptual and behavioral excesses, such as hallucinations, delusions, disorganized speech, and motor disturbances. Hallucinations [4, 5] are false sensory perceptions that occur in the absence of external stimuli and involve various sensory modalities, with auditory and visual hallucinations being the most prevalent, although olfactory, somatic, and gustatory hallucinations may occur in severe cases. Delusions [4], on the other hand, involve fixed and false beliefs that are strongly held despite contradictory evidence. Negative symptoms are marked by deficits in motivation, emotional expression, communication, and social functioning, such as avolition, anhedonia, asociality, blunted affect, and alogia [6]. These negative symptoms are more closely associated with impaired functioning, reduced quality of life, and lower productivity compared to positive symptoms [7].

The pathophysiology of schizophrenia involves complex neurobiological mechanisms, with multiple cellular pathways and neurotransmitter systems implicated. The dopaminergic system has long been central to understanding schizophrenia, with hyperactivity in dopaminergic pathways contributing to positive symptoms [8]. Other implicated systems include glutamatergic [9], serotonergic [10], cholinergic [11], and gamma-aminobutyric acid (GABAergic) pathways [12], as well as inflammatory cytokines [13].

For decades, antipsychotic medications have remained the cornerstone of schizophrenia treatment [14]. Typical (first-generation) antipsychotics primarily target dopamine D2 receptors, effectively managing positive symptoms but with limited efficacy for negative symptoms and significant adverse effects, including extrapyramidal symptoms, sedation, and metabolic disturbances. Atypical (second-generation) antipsychotics, which modulate serotonin and dopamine pathways, demonstrate improved efficacy for both positive and negative symptoms [15] while still presenting notable side effects, such as weight gain, dyslipidemia, and increased cardiovascular risk [16].

Emerging pharmacological approaches have focused on modulating alternative neurotransmitter systems. LY404039 is a potent and selective agonist for metabotropic glutamate receptors (mGluR2/3). Pomaglumetad methionil (LY2140023 monohydrate) is an oral methionine prodrug of LY404039, which acts by reducing the presynaptic release of glutamate in brain regions expressing mGlu2/3 receptors [17]. Unlike traditional antipsychotics, it does not directly interact with dopamine or serotonin (5-HT2A) receptors, potentially offering a novel mechanism of action with a different side-effect profile [18].

We aim to provide an overview of this novel drug and evaluate its efficacy in comparison to both placebo and atypical antipsychotics by performing a systematic review and meta-analysis.

## Methods

This study adhered to the Cochrane Handbook for Systematic Reviews of Interventions [19], It was reported according to the Preferred Reporting Items for Systematic Reviews and Meta-Analyses (PRISMA) statement [20], The registration number for this systematic review on PROSPERO is CRD42024614071.

### Eligibility criteria

To search for the difference between Pomglumated Methionil and antipsychotics in improving symptoms of psychotic features, we included studies according to the following PICO criteria:

1) Studies including adult schizophrenic patients.

2) Pomaglumetad Methionil was the main intervention.

3) atypical antipsychotics and/or placebo as a control.

4) The main outcomes include The Positive and Negative Syndrome Scale (PANSS) score.

5) randomized controlled trials in English.

There were no restrictions regarding the settings or the year of publication.

### Literature search

We conducted our search on multiple electronic databases including PubMed, Scopus, Web of Science, Embase, Cochrane, and Clinicaltrial.gov.

Our search strategy was **(“schizophrenia” OR “Schizoaffective disorder” OR “Psychosis” OR “Negative Symptoms of Schizophrenia” OR “psychotic disorders”) AND (“LY 2140023” OR “Pomaglumetad methionil” OR “LY2140023” OR “LY2140023 monohydrate” OR “metabotropic glutamate 2/3 receptor” OR “Metabotropic glutamate receptor” OR “mGlu2/3”)** with no filters applied.

We conducted a hand search including backward and afterward search [21]. In the backward search, we searched the listed references of included studies, while in the afterward search, we used websites such as Google Scholar to identify where each of the included studies was cited.

### Data selection and collection process

Each record was screened by two independent reviewers using Rayyan [22] we began title-abstract screening We followed it with a Full-text screening including only RCTs with 6 weeks endpoint. A third reviewer was consulted to resolve any conflict between the two authors in the inclusion decision. After full-text screening, there were 4 papers identified to be included in the study.

### Data extraction

Data extraction was performed independently by two authors using an Excel Sheet, Extracted data included: [1] the characteristics of the studies [2] Baseline characteristics of the population [3] Outcomes of interest [4] secondary outcomes and side effects.

The extracted characteristics of the studies were NCT registration code, follow-up duration, number of patients included, the interventions and comparators used, whether it was terminated or not, and the key findings.

Baseline characteristics of the population were the distribution of gender, race, country of residency, mean age, weight, BMI, total Positive and Negative Syndrome Scale (PANSS), the clinical global impression-severity scale (CGI-S).

The outcomes of interest were the Difference in total Positive and Negative Syndrome Scale (PANSS) [23] and clinical Global impression severity scale (CGI-S) [24].

The secondary outcomes were change body weight, the percentage of patients among the Standard Of Care (SOC) group that met potentially clinically significant (PCS) criteria of an increase or decrease in weight (at least 7% from baseline to endpoint), the difference in Prolactin level, PANSS positive subscale, PANSS negative subscale [25], Simpson-Angus Scale (SAS) [26], and side effects.

### Quality assessment

Each study was evaluated by two independent investigators in a premade Excel sheet using the Cochrane tool risk of bias ROB-II [27].

ROB-II has five primary domains: [1] Randomization process [2] Deviations from intended interventions [3] Bias due to missing outcome data [4] Bias in measurement of the outcome [5] Bias in selection of the reported result. Each domain is evaluated as low-risk, with some concerns or high-risk. Disagreements were resolved by discussion. An overall risk of bias assessment was made according to the following criteria:

Studies that were evaluated as a low risk of bias in all domains were judged as an overall low-risk study. Studies free of high-risk evaluated domains with at least one domain evaluated as some concerns were judged as studies with some concerns overall. Studies containing at least one domain marked as high-risk or with some concerns in multiple domains in a way that decreases the confidence in the results were judged as overall high-risk studies.

### Data synthesis

We used Mean Difference (MD) in continuous outcomes while Risk Ratio (RR) was used in binary outcomes. If an outcome was reported in multiple included studies, the outcome is considered eligible to enter the data synthesis process. Date synthesis was conducted using revman [28].

We used the chi-square test and I-square test to evaluate the heterogeneity. The I-square test was not considered significant if less than 40%, with moderate heterogeneity for 30–60%, and considered with substantial heterogeneity for 50–90%. There was considered heterogeneity if the P-value was less than 0.1. A random effect model was used to overcome heterogeneity between studies and deliver a better representation of the population. We used the inverse-variance method.

Sensitivity analysis was conducted whenever indicated.

Subgroup analysis was conducted once for the placebo group and once for the Atypical antipsychotic group. The included studies were ordered according to the year of publication, and the graphs were visualized as forest plots.

## Results

### Literature search

**Fig. 1:**
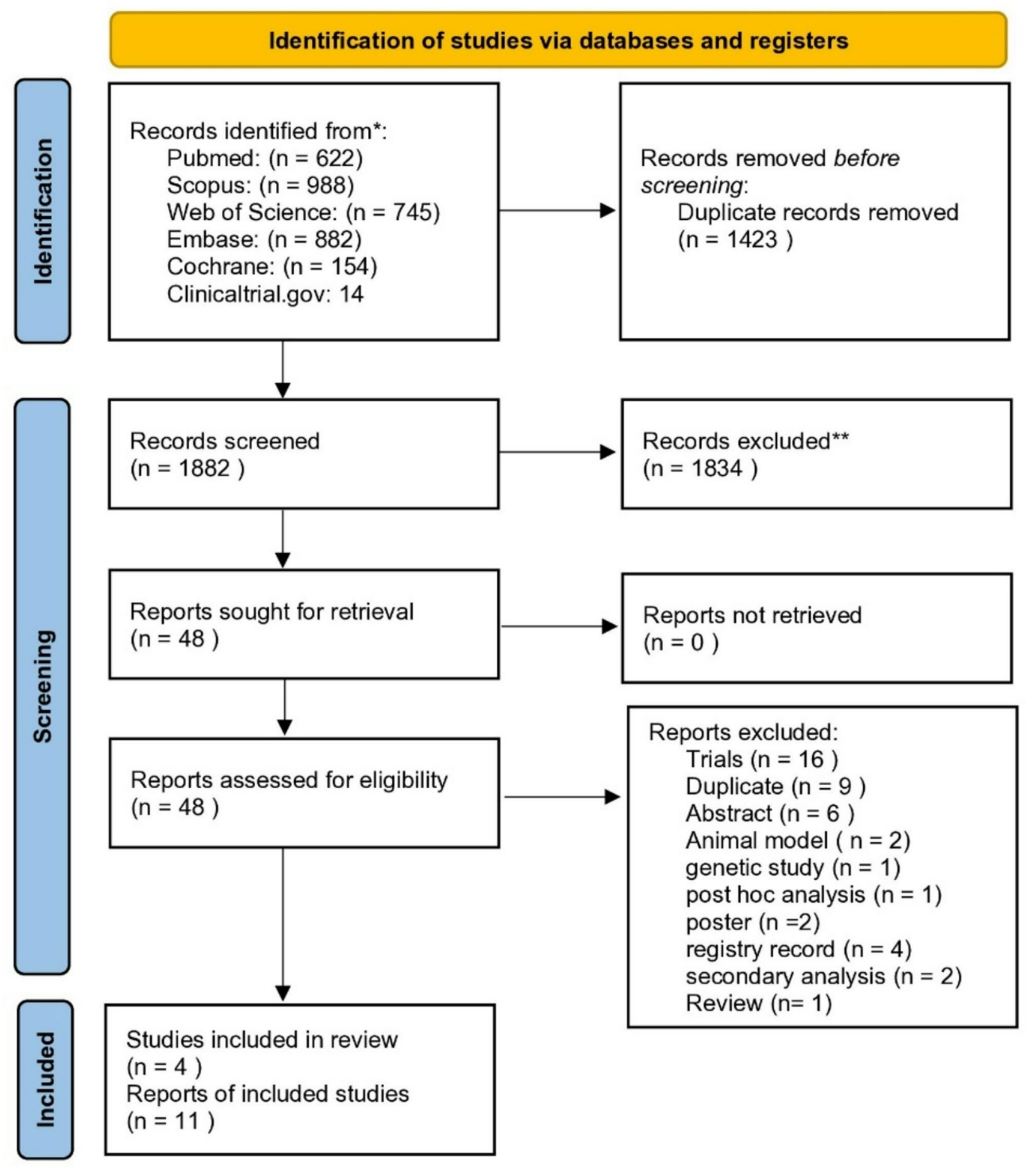
PRISMA flow diagram

We identified 3305 records through a database literature search. After removing 1423 duplicate records using Endnote, we screened 1882 through title and abstract. Among them, 1834 records did not meet eligibility criteria and were excluded. 48 were assessed after reviewing the full text. A total of 8 RCTs were included but after reviewing our inclusion criteria 4 of them didn’t meet our inclusion criteria, So finally we included 4 trials in this review as demonstrated in the PRISMA Flow diagram (Fig 1).

### Included studies

We included information from Patil [18], which is published in 2 main records and a supplementary file. Kinon [29] and Downing [30] have 1 published record and a supplementary file. We found no published record of NCT01307800 trial [31], so we included the information on the registry website. We also included information from the registry for the other included studies.

We only included the 80 mg and 160 mg doses of the drug in our review, however in our analysis we only included 80 mg dose in our metanalysis as no efficacy difference was noted between the two doses(Tables 1, 2).

**Table 1:** Study characteristics included

Study ID	*N*. Randomized	Study design	Registration number	Country	Endpoint	Intervention (s)	Comparator	Findings	Measurment
**Patil 2007**	195	Triple blind RCT	NCT00149292	Russian Federation	4 weeks	LY2140023 monohydrate 80 mg	Placebo or Olanzepine	LY2140023 monohydrate showed statistically significant improvement over placebo; However, It didn’t show a statistically significant difference compared to Olanzapine	Total PANSS score, Clinical Global Impression-Severity (CGI-S), Body weight, Prolactin level, Positive PANSS, Negative PANNS, Simpson-Angus Scale (SAS)
**Kinon 2011**	669	Double-blind RCT	EudraCT number: 2007-000800-34	Multicentric	4 weeks	LY2140023 monohydrate 10 mg, 40 mg, 80 mg, or 160 mg	Placebo or Olanzepine	LY2140023 monohydrate And olanzapine did not separate from placebo in the treatment of patients with acutely exacerbated schizophrenia.	Total PANSS score, Clinical Global Impression-Severity (CGI-S), Body weight, Prolactin level, Positive PANSS, Negative PANNS, Simpson-Angus Scale (SAS)
**NCT01307800 (2011)**	567	Double-blind RCT	NCT01307800	Multicentric	6 weeks	LY2140023 monohydrate 20, 80, 160 mg twice daily	Placebo	-	Total PANSS score, Clinical Global Impression-Severity (CGI-S), Body weight, Prolactin level, Positive PANSS, Negative PANNS, Simpson-Angus Scale (SAS)
**Downing (2017)**	1013	Double-blind RCT	NCT01086748	Multicentric	6 weeks	LY2140023 mnohydarte 80, or 160 mg	Placebo or Risperidone	LY2140023 monohydrate did not show statistically significant improvement over placebo; similarly, olanzapine did not significantly separate from placebo.	Total PANSS score

**Table 2:** Baseline characteristics of included studies

Study ID	Group	sample size	Mean Age (SD)	*N* of men (%)	Mean weight (SD)	Total PANNS MEAN SD	CGI-s Mean SD
**Patil** **(2007)**	PM 80 mg	97	39.6 (14.4)	98 (79.6%)	73.4 (12.2)	95.5 (11.4)	4.9 (0.6)
	Olanzapine	43	42.3 (13	34 (73.5%)	74.8 (12.3)	94.5 (12.9)	4.8 (0.6)
	Placebo	62	41 (1.1)	63 (77.8%)	74.1 (12.7)	93.8 (10.5)	4.9 (0.6)
**Kinon** **(2011)**	80 mg	120	38.5 (10.8)	68 (56.7)	72.5 (15.2)	99.7 (10.6)	4.9 (0.6)
	160 mg	122	36.9 (10.9)	69 (56.6%)	73.7 (15)	98.6 (13.4)	4.9 (0.6)
	Olanzapine	62	41.7 (12.3)	34 (54.8%)	73.9 (17.9)	99.6 (10)	4.9 (0.6)
	Placebo	122	38.9 (11.3)	70 (57.8%)	73 (13.4)	97.6 (12.1)	4.8 (0.7)
**NCT01307800 (2011)**	PM 80 mg	96	40.2 (11.39)	82 (74.5%)	-	-	-
	PM 160 mg	91	41.1 (11.43)	78 (70.3%)	-	-	-
	placebo	206	40.8 (11.19)	162 (71.1%)	-	-	-
**Downing (2017)**	PM 80 mg	251	39.6 (11.5)	196 (67.1%)	81.4 (20.4)	83.7 (14)	-
	PM 160 mg	229	40.5 (11.5)	183 (65.4%)	83.3 (19.2)	84.2 (14.5)	
	Risperidone	124	40.3 (11.1)	87 (61.3%)	82.7 (21.9)	84 (16.2)	-
	placebo	253	39.8 (11.4)	181 (61.4%)	81.4 (20.8)	84.3 (14.8)	-

### 3.3. Quality assessment

**Fig. 2:**
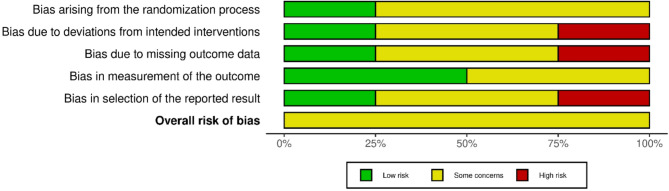
Risk of bias graph

**Fig. 3:**
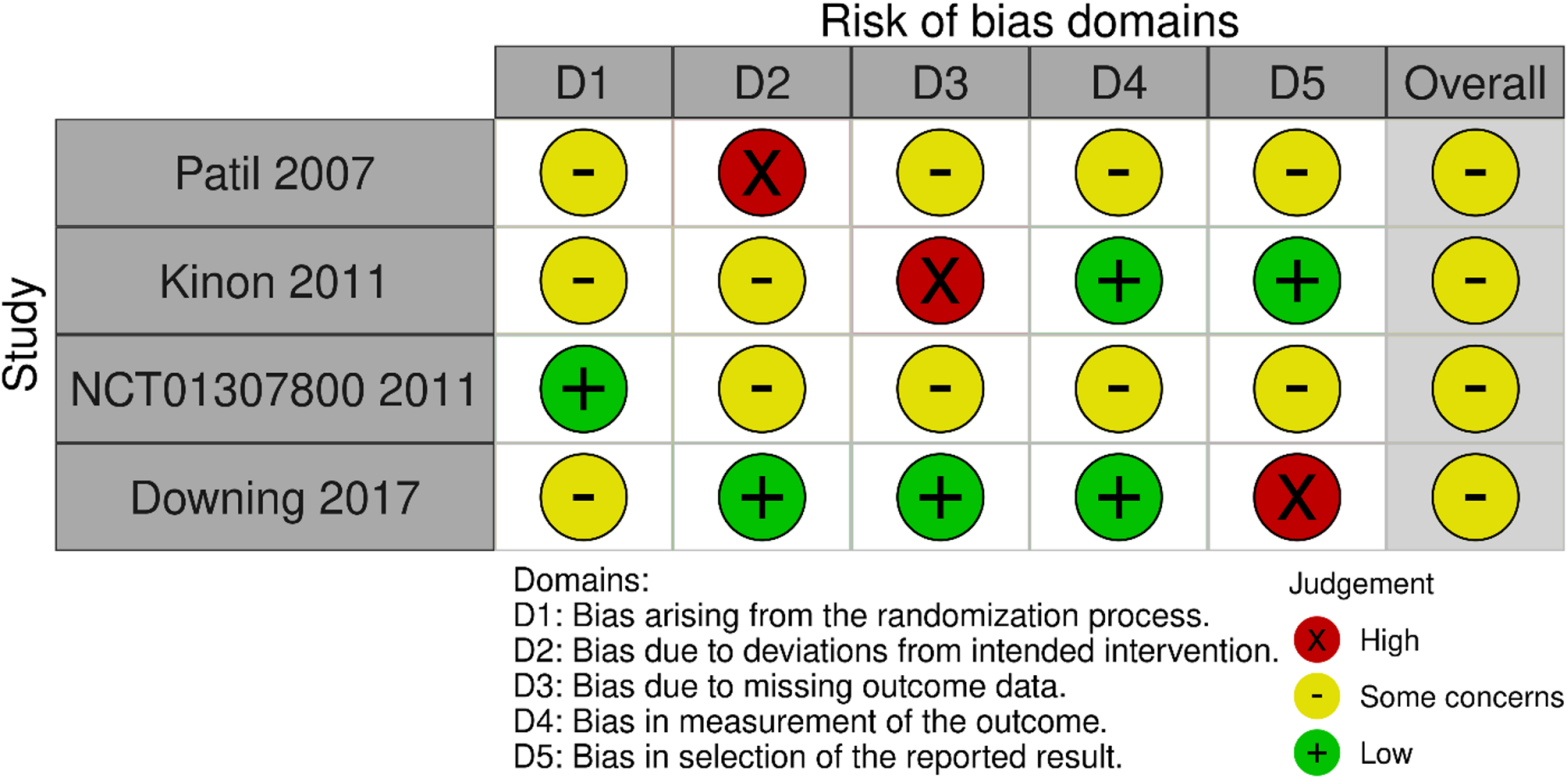
Risk of bias summary

Figure 2 and 3 (Risk of bias summary and risk of bias graph).

### Treatment efficacy on primary outcomes

#### PANSS (Positive and negative syndrome Scale)

**Fig. 4:**
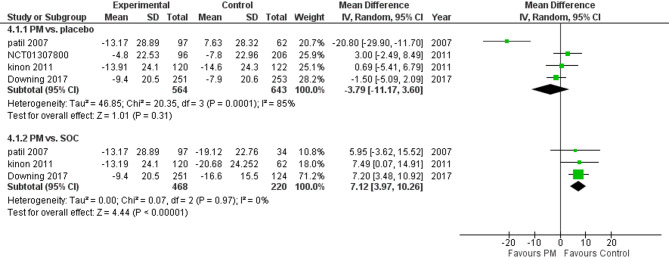
PANSS total score

**Fig. 5:**
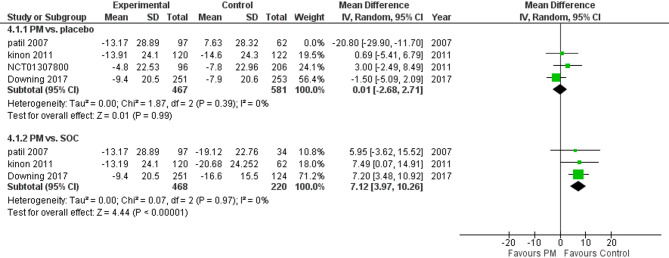
PANSS total score with sensitivity analysis

Three studies reported PANSS. We performed a subgroup analysis according to the control group and measured the mean change. The pooled analysis in PM and placebo groups showed MD = −3.79 (95% CI: −11.17 to 3.60) (Fig. 4), p-value = 0.31, indicating no significant effect. There was heterogeneity (*p* = 0.001, I² = 85%). To resolve heterogeneity, we performed sensitivity analysis by excluding Patil 2007 (*p* = 0.39, I² = 0%) (Fig. 5), however, in PM and Antipsychotics groups MD = −7.12 (95% CI: −10.26 to −3.97) (Fig. 4), p-value = 0.00001, indicating a significant effect in favor of control, with no heterogeneity (*p* = 0.97, I² = 0%).

#### CGI-S

**Fig. 6:**
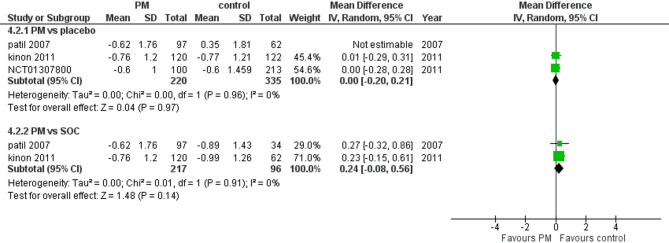
Change in CGI-s

Three studies reported CGI-S. We performed a subgroup analysis according to the control group and measured the mean change. The pooled analysis in PM and placebo groups showed MD = 0.00 (95% CI: −0.02 to 0.02) (Fig. 6), p-value = 0.97, indicating no significant effect, with no heterogeneity (*p* = 0.96, I² = 0%). However, in PM and Antipsychotics groups MD = 0.24 (95% CI: −0.08 to 0.56) (Fig. 6), p-value = 0.14, indicating no significant effect, with no heterogeneity (*p* = 0.91, I² = 0%).

### Treatment efficacy on secondary outcomes

#### Weight

**Fig. 7:**
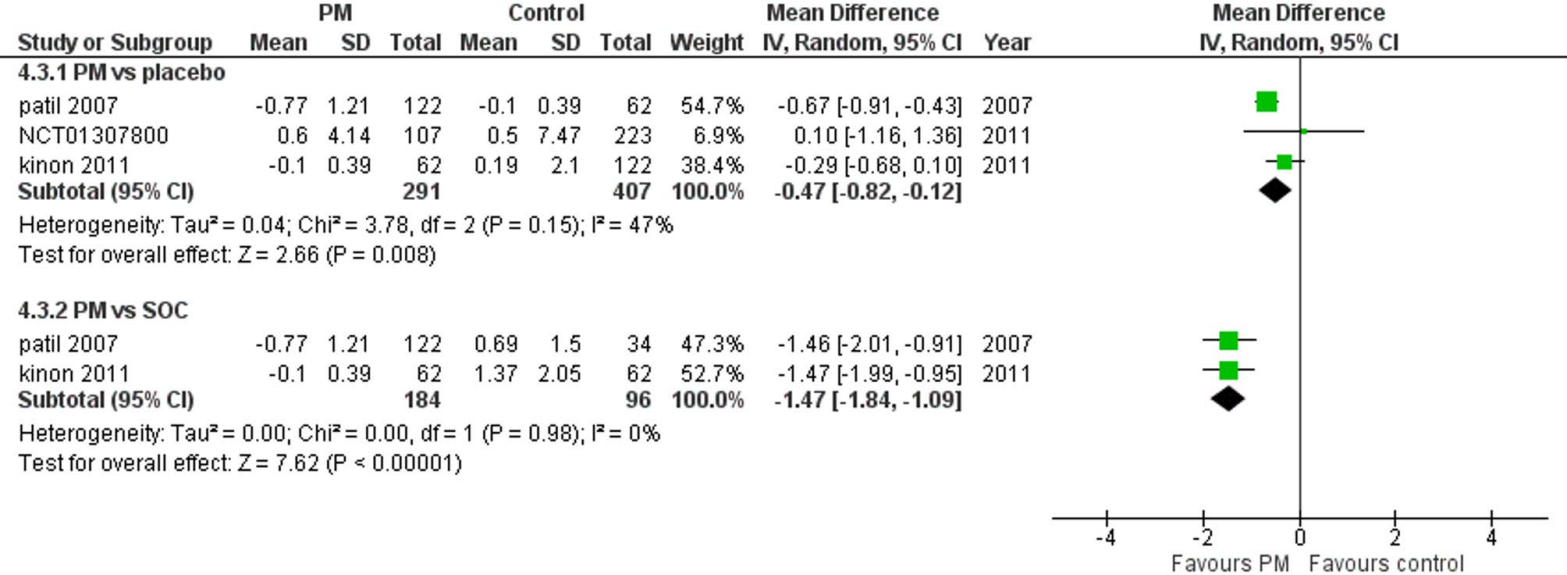
Change in body weight

Three studies reported weight. We performed a subgroup analysis according to the control group to measure the mean change. The pooled analysis between PM and placebo groups showed MD = −0.47 (95% CI: −0.82 to −0.12) (Fig. 7), p-value = 0.008, indicating a significant effect, with no heterogeneity (*p* = 0.15; I² = 47%). However, in PM and Antipsychotics groups MD = −1.47 (95% CI: −1.84 to −1.09) (Fig. 7), p-value < 0.00001, indicating a significant effect, with no heterogeneity (*p* = 0.98; I² = 0%).

#### Positive PANSS

**Fig. 8:**
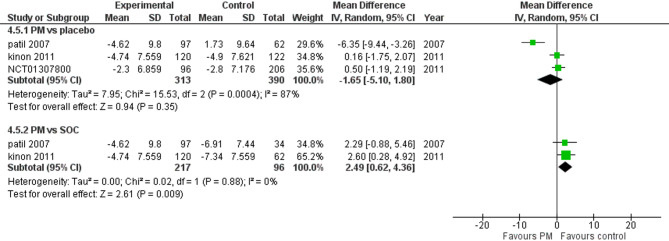
Change in panss positive subscore

**Fig. 9:**
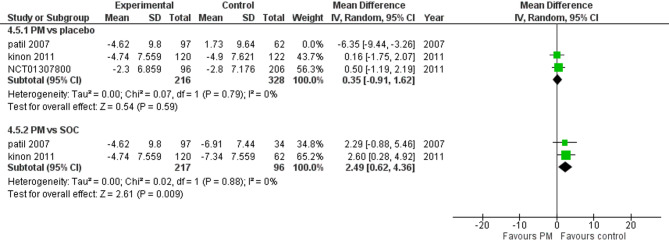
PANSS total score with sensitivity analysis

Three studies reported Positive PANSS. We performed a subgroup analysis according to the control group to measure the mean change. The pooled analysis showed MD = −1.65 (95% CI: −5.10 to 1.80) (Fig. 8), p-value = 0.35, indicating no significant effect. There was heterogeneity (*p* = 0.004; I² = 87%). To resolve heterogeneity, we performed a sensitivity test by excluding Patil 2007 (*p* = 0.79; I² = 0%) (Fig. 9), however, in PM and Antipsychotics groups MD = 2.49 (95% CI: 0.62 to 4.36) (Fig. 8), p-value = 0.009, indicating a significant effect, with no heterogeneity (*p* = 0.88; I² = 0%).

#### Negative PANSS

**Fig. 10:**
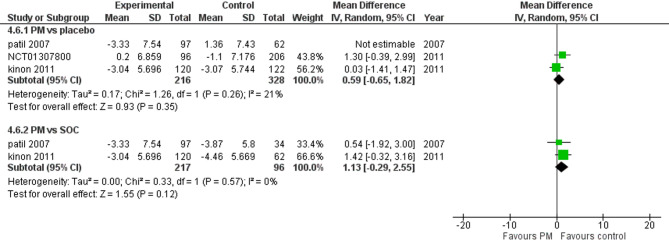
PANSS negative score

Three studies reported Negative PANSS. We performed a subgroup analysis according to the control group to measure the mean change. The pooled analysis of PM and placebo groups showed MD = −0.99 (95% CI: −3.99 to 2.01) (Fig.), p-value = 0.52, indicating no significant effect. There was heterogeneity (*p* = 0.002; I² = 88%). To resolve the heterogeneity, we performed a sensitivity test by excluding Patil 2007 (*p* = 0.26; I² = 21%) (Fig. 10), however, in PM and Antipsychotics groups MD = 1.13 (95% CI: −0.29 to 2.55) (Fig. 10), p-value = 0.12, indicating no significant effect, with no heterogeneity (*p* = 0.57; I² = 0%).

#### Prolactin

**Fig. 11:**
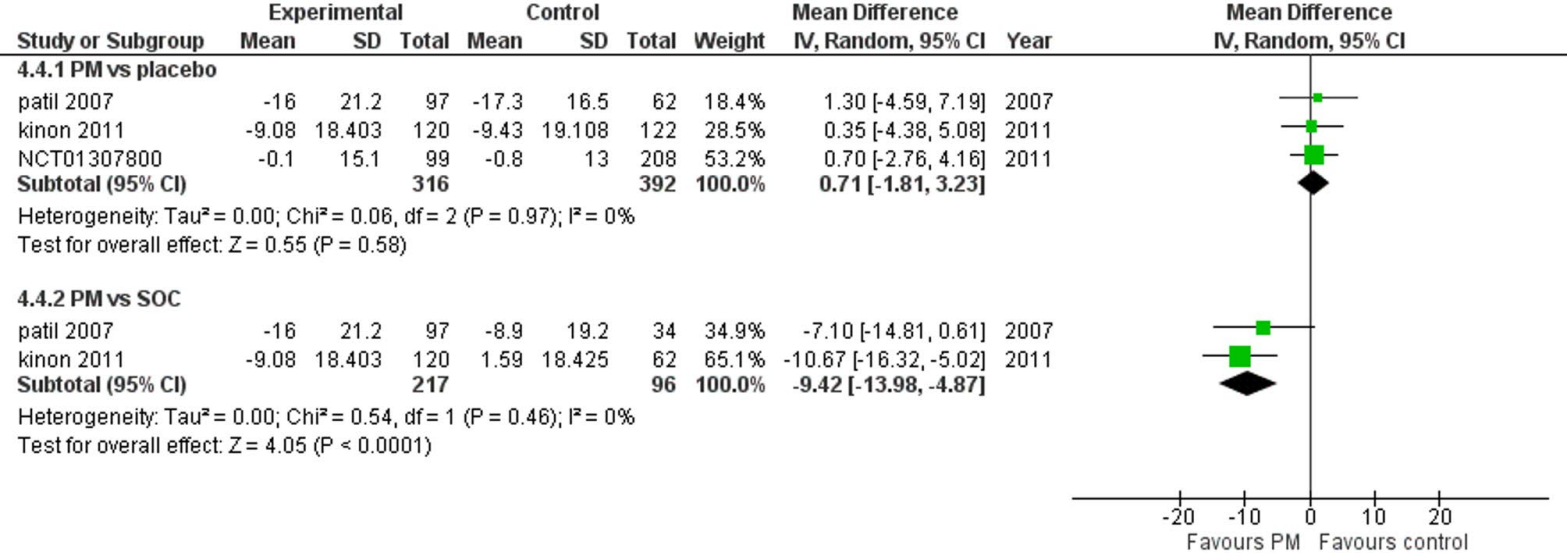
change in prolactin levels

Three studies reported prolactin. We performed a subgroup analysis according to the control group to measure the mean change. The pooled analysis of PM and placebo groups showed MD = 0.71 (95% CI: −1.81 to 3.23) (Fig. 11), p-value = 0.58, indicating no significant effect, with no heterogeneity (*p* = 0.97; I² = 0%). However, in PM and Antipsychotics groups MD = −9.42 (95% CI: −13.98 to −4.87) (Fig. 11), p-value = 0.0001, suggesting a significant effect, with no heterogeneity (*p* = 0.46; I² = 0%).

#### Simpson Angus scale (SAS)

**Fig. 12:**
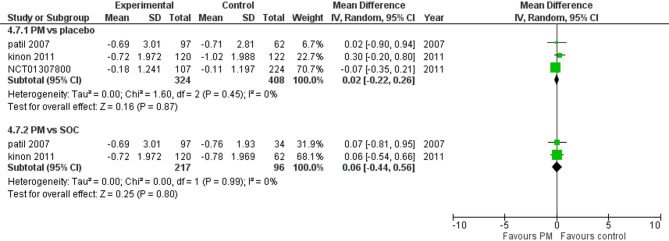
Simpson Angus Scale (SAS)

Three studies reported SAS. We performed a subgroup analysis according to the control group to measure the mean change. The pooled analysis of PM and placebo groups showed MD = 0.02 (95% CI: −0.22 to 0.26) (Fig. 12), p-value = 0.87, indicating no significant effect, with no heterogeneity (*p* = 0.45; I² = 0%). However, in PM and Antipsychotics groups MD = 0.06 (95% CI: −0.44 to 0.56) (Fig. 12), p-value = 0.80, indicating no significant effect, with no heterogeneity (*p* = 0.99; I² = 0%).

### Side effects

#### Nausea

**Fig. 13:**
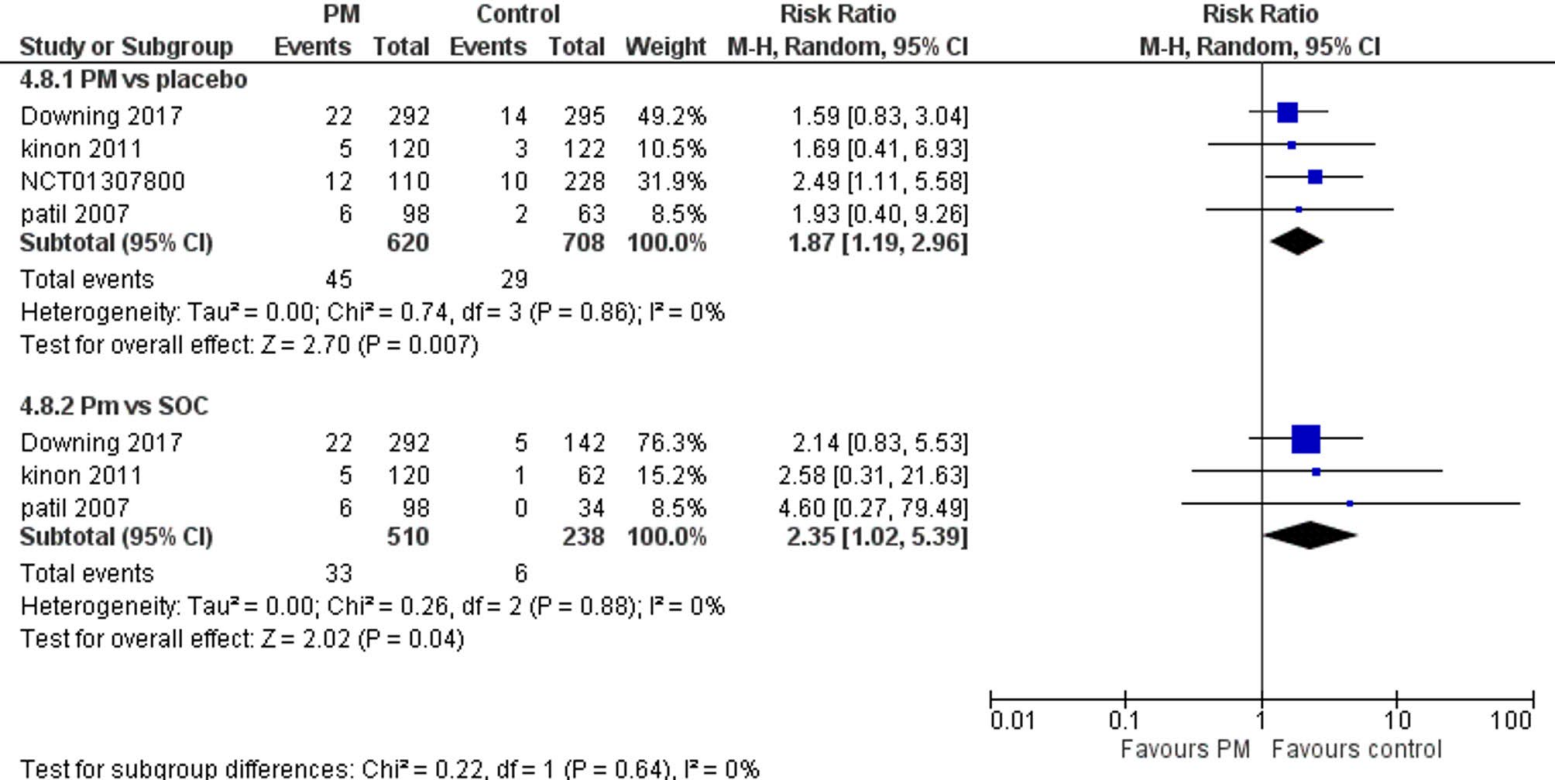
Nausea

Four studies reported nausea. We performed a subgroup analysis according to the control group to measure the risk ratio. The pooled analysis of PM and placebo groups showed RR = 1.87 (95% CI: 1.19 to 2.96) (Fig. 13), p-value = 0.007, indicating a significant effect, with no heterogeneity (*p* = 0.86; I² = 0%). However, in PM and Antipsychotics groups RR = 2.35 (95% CI: 1.02 to 5.39) (Fig. 13), p-value = 0.04, indicating a significant effect, with no heterogeneity (*p* = 0.88; I² = 0%).

#### Vomiting

**Fig. 14:**
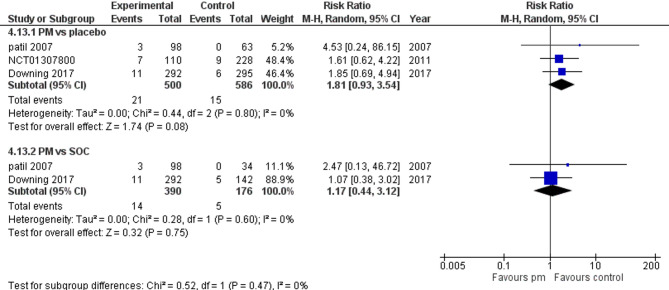
Vomiting

Three studies reported vomiting. We performed a subgroup analysis according to the control group to measure the risk ratio. The pooled analysis of PM and placebo groups showed RR = 1.81 (95% CI: 0.93 to 3.54) (Fig. 14), p-value = 0.08, indicating no significant effect, with no heterogeneity (*p* = 0.80; I² = 0%). However, in PM and Antipsychotics groups RR = 1.17 (95% CI: 0.44 to 3.12) (Fig. 14), p-value = 0.75, indicating no significant effect, with no heterogeneity (*p* = 0.60; I² = 0%).

#### Anxiety

**Fig. 15:**
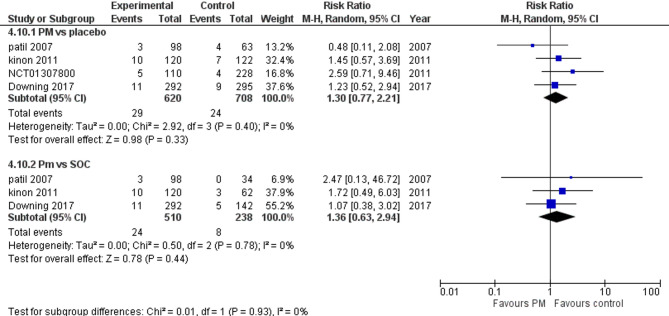
Anxiety

Four studies reported anxiety. We performed a subgroup analysis according to the control group to measure the risk ratio. The pooled analysis of PM and placebo groups showed RR = 1.30 (95% CI: 0.77 to 2.21) (Fig. 15), p-value = 0.33, indicating no significant effect, with no heterogeneity (*p* = 0.40; I² = 0%). However, in PM and Antipsychotics groups RR = 1.36 (95% CI: 0.63 to 2.94) (Fig. 15), p-value = 0.44, indicating no significant effect, with no heterogeneity (*p* = 0.78; I² = 0%).

#### Headache

**Fig. 16:**
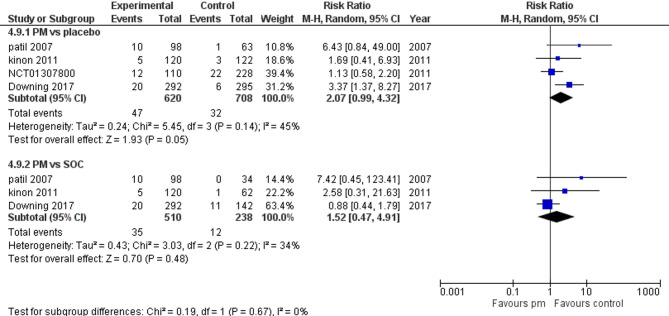
Headache

Four studies reported headaches. We performed a subgroup analysis according to the control group to measure the risk ratio. The pooled analysis of PM and placebo groups showed RR = 2.07 (95% CI: 0.99 to 4.32) (Fig. 16), p-value = 0.05, indicating a significant effect, with no heterogeneity (*p* = 0.14; I² = 45%). However, in PM and Antipsychotics groups RR = 1.52 (95% CI: 0.47 to 4.91) (Fig. 16), p-value = 0.48, indicating no significant effect, with no heterogeneity (*p* = 0.22; I² = 34%).

#### Insomnia

**Fig. 17:**
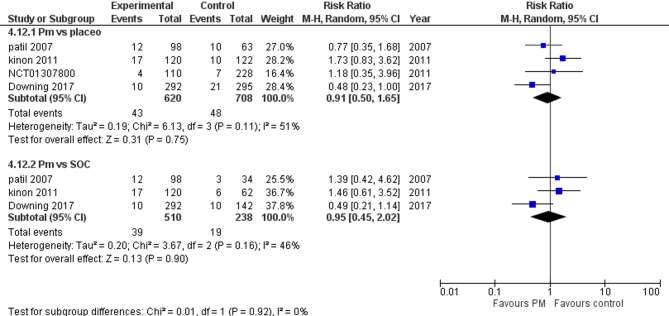
Insomnia

Four studies reported insomnia. We performed a subgroup analysis according to the control group to measure the risk ratio. The pooled analysis of PM and placebo groups showed RR = 0.91 (95% CI: 0.50 to 1.65) (Fig. 17), p-value = 0.75, indicating no significant effect, with no heterogeneity (*p* = 0.11; I² = 51%). However, in PM and Antipsychotics groups RR = 0.95 (95% CI: 0.45 to 2.02) (Fig. 17), p-value = 0.90, indicating no significant effect, with no heterogeneity (*p* = 0.16; I² = 46%).

#### Akathisia

**Fig. 18:**
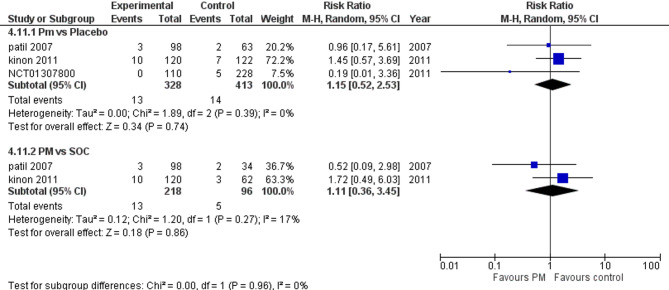
Akathasia

Three studies reported akathisia. We performed a subgroup analysis according to the control group to measure the risk ratio. The pooled analysis of PM and placebo groups showed RR = 1.15 (95% CI: 0.52 to 2.53) (Fig. 18), p-value = 0.74, indicating no significant effect, with no heterogeneity (*p* = 0.39; I² = 0%). However, in PM and Antipsychotics groups RR = 1.11 (95% CI: 0.36 to 3.45) (Fig. 18), p-value = 0.86, indicating no significant effect, with low heterogeneity (*p* = 0.27; I² = 17%).

## Result discussion

This is the first Systematic review and metanalysis of the use of promogulated methional in the treatment of schizophrenia.

Regarding the primary outcomes, the pooled analysis of PANSS scores revealed that pomaglumetad methionil (LY2140023) demonstrated a moderate but non statistically significant reduction in total PANSS scores compared to placebo in some subgroup analyses. However, the overall effect was inconsistent, with significant heterogeneity observed across studies (*p* = 0.3). Although Pomgulated methionil did decrease PANNs score when compared to atypical antipsychotics, there was noticeable difference between the two where the reduction was more noticeable in the Antipsychotics group (*p* = 0.00001), suggesting that the efficacy of pomgulated methionil is significantly less than standard antipsychotics.

Moreover, the positive PANSS subscale showed a significant difference in managing poitice symptoms in favor of Antipsychotics (*p* = 0.009), indicating that pomaglumetad methionil is less effective in addressing positive symptoms such as hallucinations and delusions in contrast to antipsychotics, which primarily target dopamine receptors and are more effective in managing positive symptoms [14]. Additionally, the negative PANSS subscale also showed no significant improvement (*p* = 0.52), suggesting that pomaglumetad methionil is not superior to placebo or antipsychotics in addressing negative symptoms.

Additionally, the analysis of CGI-S scores showed no significant difference between pomaglumetad methionil and placebo (*p* = 0.97). Similarly, there was no significant difference when compared to antipsychotics (*p* = 0.14). These results suggest that pomaglumetad methionil does not provide a clinically meaningful improvement in overall symptom severity as measured by CGI-S.

Weight gain is a major concern with traditional antipsychotics, as it can lead to metabolic syndrome and cardiovascular complications [32], Pomgulated methionil has shown potential in controlling weight gain [33], In our study pomaglumetad methionil was associated with a significant reduction in weight gain compared to SOC antipsychotics (*p* = 0.001). The favorable weight profile of this drug might suggest further research in this area.

Regarding prolactin, Pomaglumetad methionil demonstrated a significant reduction in prolactin levels compared to antipsychotics (*p* = 0.0001). This is a notable finding, as hyperprolactinemia is a common side effect of traditional antipsychotics [34] and can lead to adverse effects such as sexual dysfunction, osteoporosis, and menstrual irregularities. However, the reduction in prolactin and weight profile doesn’t compensate for the lack of efficacy of the drug; however, it might warrant further research for other safe compounds to address the needs of patients who want to avoid these side effects.

Pomaglumetad methionil was generally well-tolerated, with a side effect profile that differed from traditional antipsychotics. The most reported side effects included nausea (RR = 1.87, *p* = 0.007) and vomiting (RR = 1.81, *p* = 0.08), which were more frequent in the pomaglumetad methionil group compared to placebo. However, these side effects were generally mild to moderate in severity and did not lead to significant treatment discontinuation.

Other side effects, such as headache, anxiety, and insomnia, were not significantly different between pomaglumetad methionil and placebo. However, they tended to occur more frequently in Pomgualted methionil.

Notably, akathisia, a common side effect of traditional antipsychotics, was not significantly associated with pomaglumetad methionil (RR = 1.15, *p* = 0.74). This suggests that pomaglumetad methionil is safe for patients who are sensitive to the extrapyramidal symptoms.

## Limitations

Our study had a few limitations to address, The short follow-up period of these trials which probably takes place in the psychotic episodes might have limited our understanding of the true effect of this drug in residual state, Also the relative small sample size of these trials might have affected the scale in which the drug was studied. Although we included a good number of studies, we did exclude a few trials [33, 35, 36]of the same drugs as they were not under our inclusion criteria, some of these trials [35] studied the drugs in a longer time interval (24 weeks) however, the results showed no significant difference in comparison to our included studies.

We conducted subgroup analysis to identify possible sources of heterogeneity and few times it was detected with one study(Patil et al., 2007) being the main source of heterogeneity, to our knowledge this was the first human trial of this drug, The relatively small number of participants (157) may have contributed to this heterogeneity in these results, also Possible explanation [29] may be related to the methodological factors such as participant characteristics, trial designs and site characteristics as [18] was held in one site leading to less patient variability than other trials.

## Implications for future policy

The findings of this systematic review and meta-analysis have important implications for future policy and clinical practice. Pomaglumetad methionil (LY2140023) did not demonstrate significant efficacy compared to placebo and was less effective than atypical antipsychotics. Although it exhibited a favorable profile in terms of weight gain and prolactin levels, these safety advantages do not outweigh its limited therapeutic efficacy, the current evidence does not support the use of pomaglumetad methionil in clinical practice.

Moreover, the inconsistent efficacy observed in this review highlights the need for further research to better understand the therapeutic potential of pomaglumetad methionil, particularly in subpopulations where metabolic adverse effects are a major concern. Future studies should clarify whether specific phenotypes or stages of illness might benefit from glutamatergic modulation.

## Data Availability

All data generated or analyzed during this study are included in this published article.

## Abbreviations

PRISMA: Preferred Reporting Items for Systematic Reviews and Meta-Analyses
RCT: Randomized Controlled Trial
CI: Confidence Interval
